# Changes in the Composition of Aromatherapeutic *Citrus* Oils during Evaporation

**DOI:** 10.1155/2015/421695

**Published:** 2015-06-15

**Authors:** George W. Francis, Yen Thuy Hoang Bui

**Affiliations:** Department of Chemistry, University of Bergen, Allégaten 41, 5007 Bergen, Norway

## Abstract

The composition of some commercial *Citrus* oils, lemon, sweet orange, and tangerine, designated for aromatherapy, was examined before and after partial evaporation in a stream of nitrogen. The intact oils contained the expected mixtures of mono- and sesquiterpenes, with hydrocarbons dominating and lesser amounts of oxygenated analogues making up the remainder. Gas chromatography-mass spectrometry was used to follow alterations in the relative amounts of the various components present as evaporation proceeded. Changes were marked, and in particular more volatile components present in the intact oils rapidly disappeared. Thus the balance of content was shifted away from monoterpene hydrocarbons towards the analogous alcohols and carbonyl compounds. The results of this differential evaporation are discussed and possible consequences for aromatherapy use are noted. The case of lemon oil was especially interesting as the relative amount of citral, a known sensitizer, remaining as time elapsed represented an increasing percentage of the total oil.

## 1. Introduction

The popularity of complementary and alternative medicine has led to an increasing demand for rigorous approaches to the various methods utilised. Current work in aromatherapy is typified by the number of articles which have appeared describing the chemical composition of the various oils in common use, their benefits, and associated safety issues, and a recent book presents an excellent general introduction to this area [1]; there are also guides to their properties as relating to their application in aromatherapy [2, 3] and a survey of safety issues [4].

While much store is put on the constituents present in the various oils prior to use, little attention seems to have been directed towards the changes taking place during use. A single report discusses the alterations occurring when oils are used in an aroma lamp [5]. The question of changes during evaporation is sometimes mentioned in passing but in a way that suggests that it is of little significance. This somewhat surprising situation aroused our interest and prompted preliminary studies on mint oil to examine the extent to which differential evaporation might affect essential oil composition [6]. Following this preliminary study we decided to extend our studies to lemon (*Citrus limonum* L.) oil which is one of the most popular oils in use in aromatherapy and the results of this study are presented here. Additional investigative data was sought by examining two more* Citrus* oils, namely, those of tangerine (*C. reticulata* Blanco) and of sweet orange (*C. sinensis* L.). This investigation should be seen against the background of continuing studies into seasonal variation and new hybrids of* Citrus* species [7, 8]. The relationship between various* Citrus* species, hybrids, varieties, and cultivars is extremely complicated and the interested reader is referred to the specialised literature [9–11]. A recent review relates the difficulties that this engenders and the current state of the art with respect to analysis of these oils [12].

## 2. Material and Methods

Essential oils for aromatherapy were purchased from Plantlife (Plantlife Natural Bodycare, San Clemente, CA, USA). The oils examined were all from* Citrus* species. A preliminary gas chromatographic-mass spectrometric analysis showed that the presence and distribution of the various components within the individual oils lay within the known distributions [13]. Solvents used in preparing samples for gas chromatographic analysis were of analytical quality and used without further processing.

A very simple evaporation system was constructed using a graduated test tube of conical form into which a capillary (i.d. 0.5 mm) was directed axially. The oil under examination (1 mL) was placed in the bottom of the tube and a nitrogen gas stream at a flow rate of 2 L/min was passed through the capillary into the graduated test tube. This rate ensured that the essential oil was spread in a thin layer over the lower part of the tube to allow forced evaporation. Cooling effects were avoided by placing the test tube in a formed metal block held at 30°C. Samples were examined prior to evaporation and after volume reductions of 50 ± 4% (about 25 min) and 90 ± 2% (about 45 min).

A gas chromatograph 6090N from Agilent was connected to a JEOL AccuTOF JMS-T100GC mass spectrometer. The GC column utilised was a highly inert nonpolar Agilent VF-1MS column of 25 meters, 200 *μ*m inner diameter, and film thickness 0.33 *μ*m. Helium pressure was 81.8 kPa and rate 0.7 mL/min, with a split/splitless injector. Samples for analysis (0.2 *μ*L) were injected using a 1 : 50 split. Temperature program was as follows: initial temperature 50°C, heating rate of 30°C/min from 50 to 150°C, 8°C/min from 150 to 300°C, and finally 5 min isothermally at 300°C.

Mass spectra were initially identified using inbuilt software and library spectra (NIST) and identities were then confirmed by individual manual inspection. While identifications should be regarded as tentative further evidence was accrued by comparison of the order of elution with published chromatograms of the corresponding oils [13] and examining available data for boiling points.

## 3. Results and Discussion

**Figure 1:**
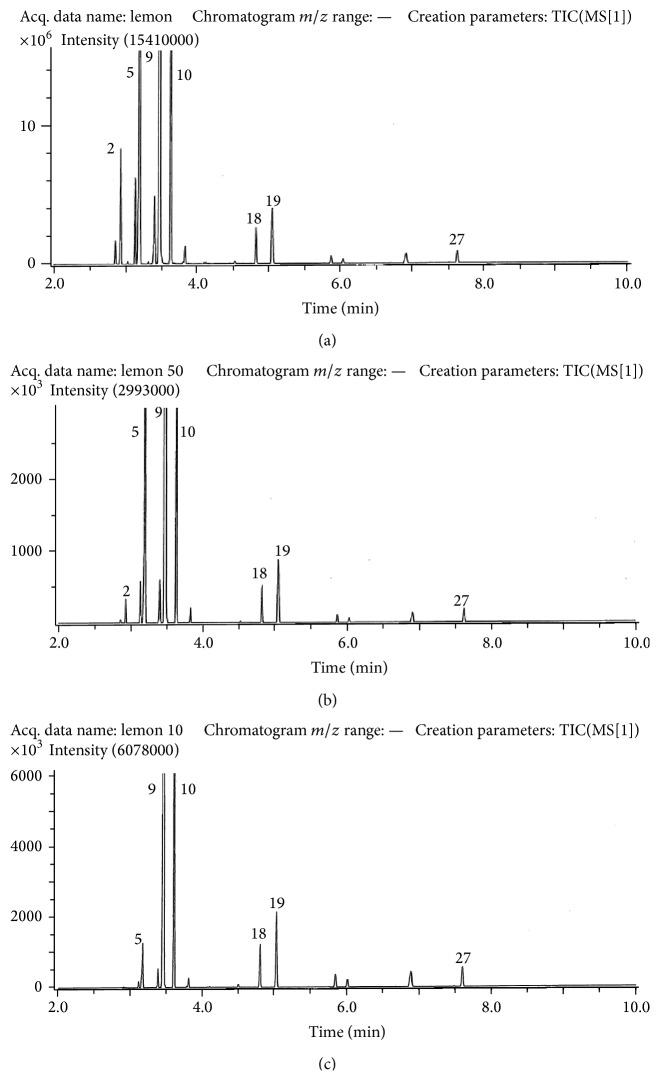
Gas chromatograms obtained for lemon oil. Upper: before evaporation; middle: after 50% evaporation; and bottom: after 90% evaporation. Conditions are as described in the experimental part. Compounds are numbered as in Table 1.

**Figure 2:**
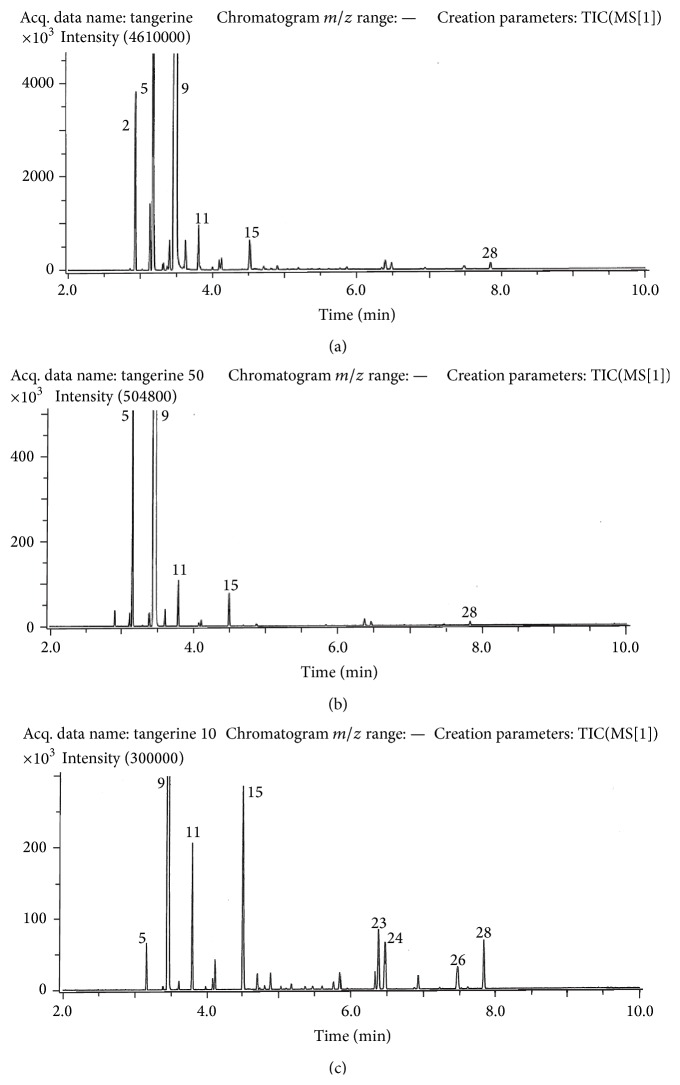
Gas chromatograms obtained for tangerine oil. Upper: before evaporation; middle: after 50% evaporation; and bottom: after 90% evaporation. Conditions are as described in the experimental part. Compounds are numbered as in Table 1.

**Figure 3:**
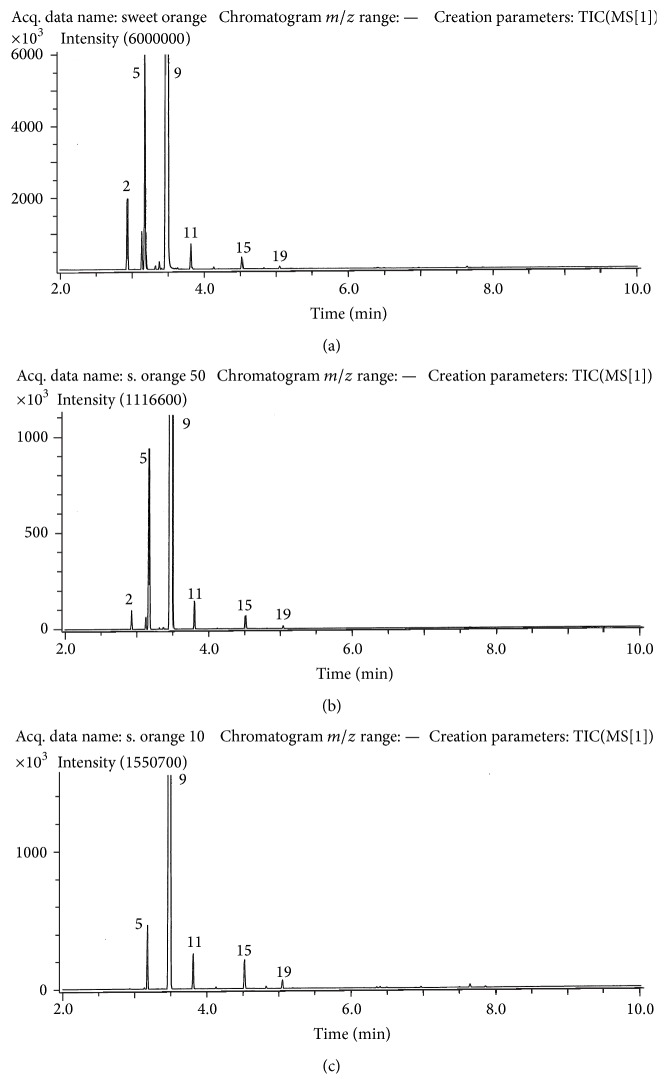
Gas chromatograms obtained for sweet orange oil. Upper: before evaporation; middle: after 50% evaporation; and bottom: after 90% evaporation. Conditions are as described in the experimental part. Compounds are numbered as in Table 1.

The results follow the same pattern in a general way for each of the oils, and gas chromatograms are presented in Figures 1–3. The column used for separation was of nonpolar type and the elution order thus followed the boiling points of the compounds. Compounds are identified in Table 1 which also provides percentage compositions based on peak volumes. The main compound in each of the oils was limonene (9) with increasing amounts as one progressed along the series from lemon to tangerine and sweet orange. A change in the opposite sense was found for *β*-pinene (5).

The composition of the lemon oil was dominated by monoterpene hydrocarbons and contained in addition to limonene (9) and *β*-pinene (5) a considerable amount of *γ*-terpinene (10). Smaller amounts of the further monoterpenes 2-thujene (1), *α*-pinene (2), camphene (3), *β*-phellandrene (4), o-cymene (8), and terpinolene (12) were also registered. The remaining compounds were oxygenated monoterpenoids or sesquiterpenes. Examining the changes taking place on evaporation it is quite clear that there is a severe reduction in the less polar constituents which elute prior to limonene (9). The oxygenated compounds, in particular *β*-citral (18) and *α*-citral (19), and sesquiterpenes show relatively increasing amounts as the elapsed time and thus evaporation increase.

The changes taking place as evaporation takes place in tangerine (Figure 2) follow the pattern already seen in lemon. The main compounds of the intact tangerine oil are *α*-pinene (2), *β*-pinene (5), and limonene (9). During evaporation the amounts of pinenes are drastically reduced while the relative importance of *β*-linalool (11) and decanol (15) are much increased. The sesquiterpenes present also make a larger contribution to the mixture as time elapses.

The final oil examined was that of sweet orange. This oil is very largely dominated by limonene (9) which represents well 95% of the total in the intact sample. The lesser amounts of the pinenes (2 and 5) make up most of the remainder. As evaporation advances (Figure 3) the dominance of limonene (9) increases and is almost total when only 10% of the oil remains.

Taken together the results show very clearly that evaporation leads to drastic changes in the content of the remaining material and this indicates the changing nature of the evaporated material as time elapses. The general effects are the rapid removal of the monoterpene hydrocarbons, particularly those more volatile than limonene (9), and the gradual increase in the relative amounts of oxygenated compounds and sesquiterpenes.

The results above clearly demonstrate the effects of evaporation with time. While the extent to which this differential evaporation occurs will depend on the equipment being used it is clearly a problem that deserves consideration. In particular, since the effects of individual compounds in aromatherapy treatment are believed to differ [2], the treatment being applied to the patient will differ as the individual session is carried out. The changes taking place resulting from differential evaporation may prove to be confounding with respect not only to individual components, but also to synergic effects.

The case of lemon oil provides an example of the inherent dangers that may result from differential evaporation. The EU requires the labelling of 26 fragrances because of sensitization effects and three of these, citral, limonene, and linalool, occur in lemon oil [14]. Citral is classified as a group II allergen, while limonene and linalool are classified as less potent group III allergens. Our results indicate more than doubling of the relative amounts of citral in lemon oil during evaporation, and thus a corresponding danger for sensitisation as evaporation proceeds.

## 4. Conclusions

It is apparent that if results of aromatherapy are to be consistent it is important to avoid the effects of differential evaporation. Our own experiments and those reported for the use of aroma lamps show the inherent difficulties to be met. The problems to be met will clearly depend on the aromatherapy method being utilised. The most severe effects are expected to be greatest when air streaming or forced drafting are used to provide aromatic atmospheres, although any method involving evaporation may be affected. While the use of aerosols can reduce or totally avoid eventual uncertainties, the production of aerosols is a matter of considerable difficulty and the use of nebulisers, even when specially designed for medicinal purposes, is complicated, and the interested reader is referred to a recent review for an oversight of the situation [15].

## 5. Highlights

 GC-MS of* Citrus* oils.
 Changes in composition during evaporation.
 Consequences of differential evaporation for aromatherapy.

## Figures and Tables

**Table 1:** Percentage composition of *Citrus* oils as determined by GC-MS. Numbers in parenthesis after chemical names refer to peak numbers on chromatograms. Oils are identified as lemon, tangerine, and sweet orange. Column heads show percentage of oil remaining at analysis point. GC was carried out under the following conditions: VF-1MS column of 25 meters × 200 *μ*m (i.d.), film thickness 0.33 *μ*m, at helium pressure 81.8 kPa, and rate 0.7 mL/min, with a split/splitless injector. Samples for GC-MS were analysed after dilution 1 : 10 (v : v) and an aliquot (0.2 *μ*L) was injected with a 1 : 50 split. Temperature program: initial temperature 50°C, with immediate heating rate of 30°C/min from 50 to 150°C, 8°C/min from 150 to 300°C, and finally 5 min isothermal at 300°C.

Compound (peak numbers)	Essential oils								
	Lemon			Tangerine			Sweet orange		
	100%	50%	10%	100%	50%	10%	100%	50%	10%
2-Thujene (1)	0.5	0.1							
*α*-Pinene (2)	2.7	0.7		3.2	0.1		1.1	0.2	
Camphene (3)	0.1								
*β*-Phellandrene (4)	2.4	1.3		1.2	0.1		0.5	0.1	
*β*-Pinene (5)	17.5	9.8	2.9	8.5	2.4	0.2	3.0	2.8	1.1
*α*-Phellandrene (6)							0.1		
3-Carene (7)							0.1		
o-Cymene (8)	1.9	1.5	1.8	0.5	0.1				
Limonene (9)	58.6	61.7	61.0	84.3	95.0	96.9	94.6	96.1	97.8
*γ*-Terpinene (10)	11.7	19.4	22.7	0.4	0.3				
*β*-Linalool (11)				0.5	0.7	0.7	0.4	0.4	0.5
Terpinolene (12)	0.3	0.3	0.5						
*cis*-Mentha-2,8-dien-1-ol (13)				0.1	0.1				
Isopulegol (14)				0.1	0.1	0.1			
Decanal (15)				0.4	0.5	0.9	0.2	0.3	0.4
*α*-Terpineol (16)	0.1	0.1	0.2						
cis-Carveol (17)				0.1	0.1				
*β*-Citral (18)	1.1	1.3	2.7	0.1	0.1				
*α*-Citral (19)	2.0	2.2	4.8					0.1	0.2
D-Carvone (20)				0.1	0.1				
Nerol (21)	0.3	0.4	0.7						
Neryl acetate (22)	0.1	0.2	0.4						
*α*-Copaene (23)				0.2	0.2	0.3			
*β*-Copaene (24)				0.1	0.1	0.2			
*α*-Bergamotene (25)	0.3	0.4	1.0						
Germacrene D (26)				0.1	0.1	0.1			
*β*-Bisabolene (27)	0.4	0.6	1.3						
*δ*-Cadinene (28)				0.1	0.1	0.2			
